# Cutaneous larva migrans with optic disc edema: a case report

**DOI:** 10.1186/1752-1947-4-209

**Published:** 2010-07-07

**Authors:** Luna Dhir, Tim O'Dempsey, Mark T Watts

**Affiliations:** 1 West of England Eye Unit, Royal Devon and Exeter Hospital, Exeter, UK; 2 Liverpool School of Tropical Medicine, Liverpool, UK; 3 Department of Ophthalmology, Arrowe Park Hospital, Wirral, UK

## Abstract

**Introduction:**

A rare case of optic disc edema associated with cutaneous larva migrans is presented. To the best of our knowledge, this has not been previously reported in literature. Joint management by ophthalmology and tropical medicine teams proved most beneficial for our patient, facilitating correct diagnosis, appropriate investigations and instigation of suitable treatment.

**Case presentation:**

A 45-year-old Caucasian man, a naturalist, from the UK developed cutaneous larva migrans while in Kenya and presented to us with visual disturbance secondary to unilateral optic disc edema. This resolved after receiving a single dose of ivermectin and visual acuity reverted to normal.

**Conclusion:**

To the best of our knowledge, optic disc edema associated with cutaneous larva migrans has not been previously reported. This case highlights the importance of taking relevant history of recent travel to endemic areas affected by the nematodes in patients presenting with optic disc edema, and pertinent questioning regarding non-ocular symptoms, including skin lesions. In this case, a history of recent foreign travel and treatment for skin lesions was crucial.

## Introduction

Optic disc edema associated with cutaneous larva migrans is an unusual presentation in the UK, although diffuse unilateral sub-acute neuroretinitis (DUSN) caused by a motile sub-retinal nematode has been reported in the USA, the Caribbean Islands, Brazil, Germany, Venezuela, Canada and China [1]. We report a case of cutaneous larva migrans associated with unilateral optic disc edema, which resolved after a single dose of ivermectin.

## Case presentation

A 45-year-old Caucasian man from the UK was referred by his general practitioner to the ophthalmic casualty department, with a four-week history of blurred vision and floaters in the left eye, pain on ocular movement and left-sided headache. There was no previous ophthalmic history and his general health was good. Being a naturalist, our patient had been on a two-week expedition to mangrove swamps in Kenya, and had returned six weeks ago. His ocular symptoms developed two weeks after his return from Kenya.

He gave a history of being referred to a School of Tropical Medicine by his general practitioner on his return, as he had developed skin lesions. He was seen there by appointment five weeks after his return (a week prior to presentation at the eye clinic). He was diagnosed to have cutaneous larva migrans and was treated by the tropical medicine specialist with a single oral dose of 18 mg ivermectin. The ocular symptoms preceded the treatment with ivermectin by three weeks.

**Figure 1:**
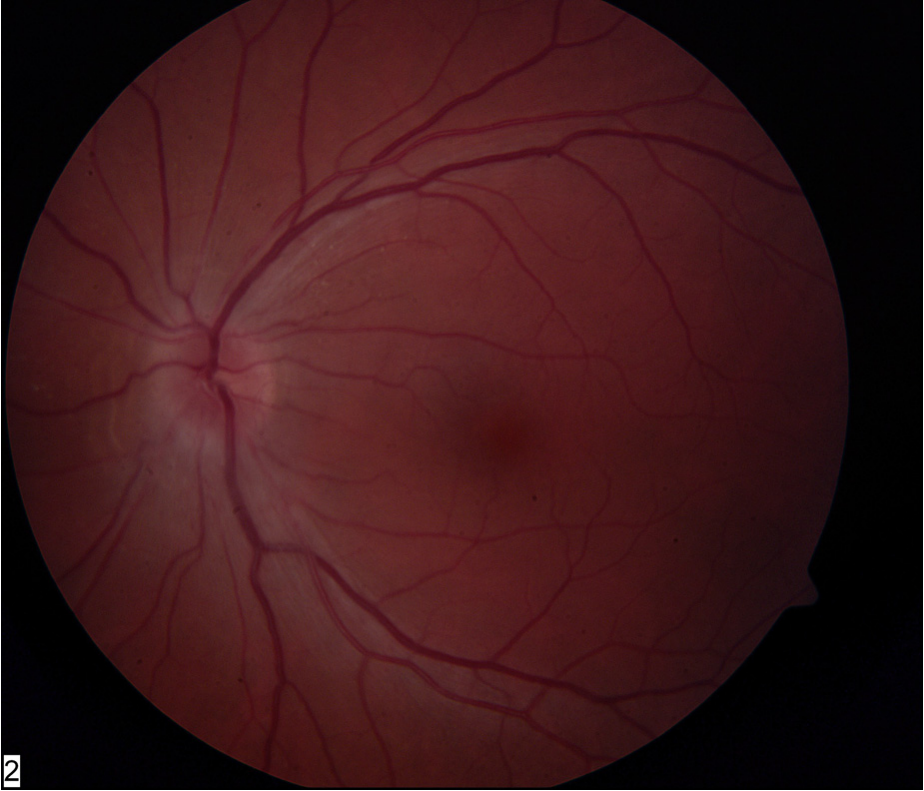
**Appearance of the left optic disc on presentation (swollen)**.

Visual acuities were 6/6 in both eyes. Anterior segment, pupillary reflexes, color vision, Pelli Robson contrast sensitivity and Humphrey visual fields were normal. Fundus examination showed the presence of a swollen optic disc in the left eye (Figure 1). The right optic disc was normal.

Physical examination revealed the presence of typical lesions of cutaneous larva migrans on arms, legs and back. Blood investigations including inflammatory markers and auto antibodies were normal. Serum was negative for syphilis, toxoplasmosis, toxocara and brucellosis. Magnetic resonance imaging (MRI) of orbits and brain was normal.

**Figure 2:**
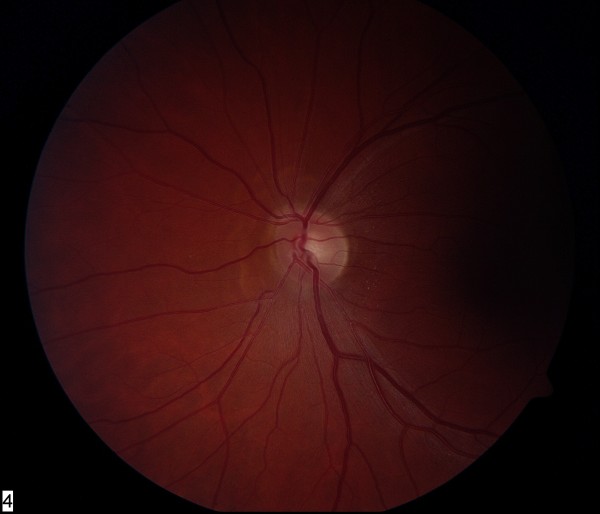
**Resolution of left optic disc edema after treatment with ivermectin**. Low magnification.

**Figure 3:**
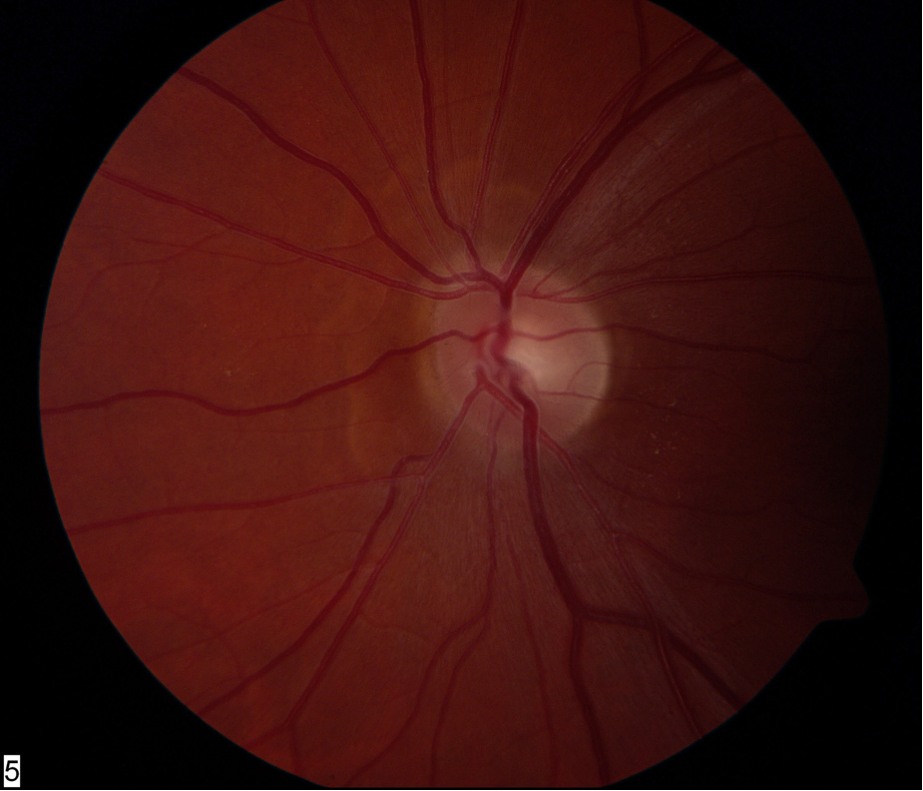
**Resolution of left optic disc edema after treatment with ivermectin**. High magnification.

Visual symptoms, skin lesions and optic disc edema resolved over the course of two to three weeks. On last review, visual acuity was 6/6 in both eyes, color vision and visual fields were normal and there was complete resolution of disc edema (Figures 2 and 3).

## Discussion

To the best of our knowledge, no case of cutaneous larva migrans associated with isolated optic disc edema has been reported in literature. The authors feel this may be a case of optic perineuritis (peri-optic neuritis). On the other hand, diffuse unilateral sub-acute neuroretinitis (DUSN) is well described.

DUSN is an ocular infectious disease caused by either of two different sized and as yet unidentified nematodes capable of causing profound visual loss in one or both eyes. The smaller nematode measures 500 to 600 microns in length and is likely to be *Ancylostoma caninum*, the dog hookworm and common cause of cutaneous larva migrans or the larval form of *Toxocara canis *[2]. The larger nematode measures 1000 to 2000 microns and is believed to be *Baylisascaris procyonis*, the raccoon roundworm. DUSN causes uniocular persistent vitritis associated with papillitis, retinal vasculitis and multifocal lesions involving the outer retinal layers. In late stages, optic disc and retinal atrophy may occur. Oral high dose albendazole [1,3], thiabendazole or a single oral dose of ivermectin [4] has been used for effective anti-helminthic therapy. Alternatively, the nematode can be directly destroyed with argon laser photocoagulation. A scanning laser ophthalmoscope has been used to assist identification of the live nematode [5].

We believe the causative organism in our patient with optic disc edema is likely to be *A. caninum *due to the fact that he had classic lesions of cutaneous larva migrans and as other investigations were normal. We can speculate that an *A. caninum *larva migrated into or close to the optic nerve, causing perioptic neuritis. The ocular symptoms preceded systemic treatment with ivermectin, and therefore cannot be drug-induced. There were no other findings of DUSN and the clinical course was good, with no involvement of the retinal vasculature or outer retina. Although he presented late, cure was achieved with a single dose of oral ivermectin.

## Conclusions

This case highlights the importance of taking relevant history of recent travel to endemic areas affected by the nematodes in patients presenting with optic disc edema, and pertinent questioning regarding non-ocular symptoms, including skin lesions. In this case, history of recent foreign travel and treatment for skin lesions was crucial.

The case also emphasizes appropriate liaison with relevant experts in management of unusual cases.

## Competing interests

The authors declare that they have no competing interests.

## Authors' contributions

LD was involved in the care of the patient, drafting and writing the manuscript and literature review. TO was involved with the treatment and investigations. MTW critically revised the manuscript. All authors read and approved the final manuscript.

## Consent

Written informed consent was obtained from the patient for publication of this case report and any accompanying images. A copy of the written consent is available for review by the Editor-in-Chief of this journal.
